# Hypomethylating agent and venetoclax with FLT3 inhibitor “triplet” therapy in older/unfit patients with *FLT3* mutated AML

**DOI:** 10.1038/s41408-022-00670-0

**Published:** 2022-05-02

**Authors:** Musa Yilmaz, Hagop Kantarjian, Nicholas J. Short, Patrick Reville, Marina Konopleva, Tapan Kadia, Courtney DiNardo, Gautam Borthakur, Naveen Pemmaraju, Abhishek Maiti, Elias Jabbour, Nitin Jain, Ghayas Issa, Koichi Takahashi, Koji Sasaki, Maro Ohanian, Sherry Pierce, Guillin Tang, Sanam Loghavi, Keyur Patel, Sa A. Wang, Guillermo Garcia-Manero, Michael Andreeff, Farhad Ravandi, Naval Daver

**Affiliations:** 1 Department of Leukemia, The University of Texas MD Anderson Cancer Center, Houston, USA; 2 Department of Hematopathology, The University of Texas MD Anderson Cancer Center, Houston, USA

**Keywords:** Medical research, Health care

## Abstract

In older/unfit newly diagnosed patients with *FLT3* mutated acute myeloid leukemia (AML), lower intensity chemotherapy (LIC) in combination with either a FLT3 inhibitor or with venetoclax results in poor overall survival (median 8 to 12.5 months). We performed a retrospective analysis of 87 newly diagnosed *FLT3* mutated AML patients treated on triplet (LIC + FLT3 inhibitor + Venetoclax, [*N* = 27]) and doublet (LIC + FLT3 inhibitor, [*N* = 60]) regimens at our institution. Data were collected from prospective clinical trials in 75% (*N* = 65) and 25% (*N* = 22) who received the same treatment regimens outside of a clinical trial. Triplet therapy was associated with significantly higher rates of complete remission (CR) (67% versus 32%, *P* = 0.002), CR/CRi (93% versus 70%, *P* = 0.02), FLT3-PCR negativity (96% versus 54%, *P* < 0.01), and flow-cytometry negativity (83% versus 38%, *P* < 0.01) than doublets. At the end of the first cycle, the median time to ANC > 0.5 (40 versus 21 days, *P* = 0.15) and platelet > 50 K (29 versus 25 days, *P* = 0.6) among responders was numerically longer with triplets, but 60-day mortality was similar (7% v 10%). With a median follow-up of 24 months (median 12 months for triplet arm, and 63 months for doublet arm), patients receiving a triplet regimen had a longer median overall survival (not reached versus 9.5 months, *P* < 0.01). LIC combined with FLT3 inhibitor and venetoclax (triplet) may be an effective frontline regimen for older/unfit *FLT3* mutated AML that should be further validated prospectively.

## Introduction

Internal tandem duplication (ITD) and tyrosine kinase domain (TKD) mutations in the *FLT3* gene are some of the most common mutations in patients with newly diagnosed acute myeloid leukemia (AML). *FLT3-*ITD mutations are associated with a higher risk of relapse and inferior overall survival (OS) [1, 2]. Midostaurin, a first-generation FLT3 inhibitor, combined with intensive chemotherapy, improved OS in younger adult patients (<60 years) with *FLT3* mutated AML [3]. However, adding a first-generation FLT3 inhibitor (sorafenib or midostaurin) to azacitidine in older adults who are not fit for intensive therapy provided modest outcomes. CR/CRi rates were 78% and 38%, and median OS was 8.3 and 8.7 months with azacitidine with sorafenib and azacitidine with midostaurin, respectively, in frontline older/unfit patients [4, 5].

In a recently published phase I/II study of azacitidine in combination with second-generation FLT3 inhibitor, quizartinib, a small cohort of older/unfit patients with newly diagnosed *FLT3* mutated AML had a CR/CRi rate of 87% and median OS of 19 months, suggesting combining a second-generation FLT3 inhibitor with azacitidine may improve outcomes [6]. Unfortunately, the LACEWING phase III, randomized, open-label study of azacitidine with or without gilteritinib was stopped at the interim analysis due to futility [7]. Despite CR/CRi rate being improved with azacitidine with gilteritinib compared with azacitidine alone (74% versus 41%, *P* < 0.001), the median OS was similar in both arms (9.8 versus 8.9 months, *P* = 0.75), postulated to be due to a higher frequency of subsequent salvage therapy and an especially higher proportion of salvage FLT3 inhibitor use (including gilteritinib) in the patients randomized to azacitidine alone. On a post-hoc subset analysis, patients with a higher *FLT3* allelic ratio (AR) appeared to benefit from azacitidine and gilteritinib [7]. These findings have somewhat dampened enthusiasm to use a hypomethylating agent (HMA) with FLT3 inhibitor doublets as frontline therapy for older/unfit AML.

After demonstrating a median OS of approximately 15 months in older/unfit patients with newly diagnosed AML in the VIALE-A study, HMA with venetoclax has emerged as a new standard of care for this population [8]. Despite a CR/CRi rate of 67%, the median OS in *FLT3* mutated patients with frontline HMA plus venetoclax in a pooled analysis of the phase IB and phase III VIALE-A study was only 12.5 months, lower than what has been achieved in non-*FLT3* mutated patients with this regimen suggesting that HMA with venetoclax may not be a sufficiently effective therapy in these patients [9].

MCL-1 and BCL-XL are overexpressed in venetoclax resistant AML cells [10, 11]. Primary and secondary resistance to venetoclax can be mediated by pre-existing or emergent *FLT3*-ITD mutations [12, 13], and FLT3 inhibition may increase BCL-2 dependency by concurrently downregulating anti-apoptotic protein MCL1, thereby increasing sensitivity to venetoclax [14]. Numerous preclinical studies have demonstrated synthetic lethality when venetoclax was combined with a FLT3 inhibitor [15–17]. Clinically the combination of venetoclax with gilteritinib has shown robust activity with composite complete remission (CRc) rates of >75% and FLT3 molecular response (<10^−2^) in 60% of the responders, with response rates being maintained in prior FLT3 inhibitor exposed patients [18]. In a recently published clinical study by our group, a small cohort of older/unfit patients with newly diagnosed *FLT3* mutated AML were treated with HMA, venetoclax, and FLT3 inhibitor combination (triplet regimen) on a clinical trial. Of the 12 patients who received the triplet regimen, 11 (92%) achieved a CRc with measurable residual disease (MRD) negativity by FLT3-PCR in 91% of the CRc patients [19]. In this current manuscript, we analyze a larger cohort of older/unfit patients with newly diagnosed *FLT3* mutated AML (including the 12 cases reported previously [19]) treated with a triplet regimen (low-intensity chemotherapy + FLT3 inhibitor + venetoclax) and compare the CR and CRc rates, MRD dynamics, count recovery kinetics, early mortality, and OS with patients who received a doublet regimen (low-intensity chemotherapy + FLT3 inhibitor) at our institution within the last decade.

## Methods

### Patient Eligibility

We identified 87 older and or unfit (for intensive chemotherapy) adult patients with newly diagnosed *FLT3* mutated (ITD and or TKD) AML treated at our institution between June 2012 to March 2021 with FLT3 inhibitor-based LIC regimens. All patients had at least two or more bone marrow (BM) assessments, including baseline, end of the first cycle of therapy, and/or later during treatment. MRD assessments were performed by multicolor flow cytometry (MFC, sensitivity of 10^−3^)[20] and multiplex polymerase chain reaction (FLT3-PCR, sensitivity of 10^−2^) for ITD and kinase domain (D835), as previously published by our group [21]. The best MRD was defined as negative or lowest MRD level achieved at any time during frontline therapy prior to the last follow-up (in CRc patients), prior to relapse, and prior to allogeneic stem cell transplant (ASCT). Any level of detectable MRD was called positive. Next-generation sequencing was performed using one of three myeloid gene panel (81-gene, 53-gene, 28-gene) platforms validated and implemented at our institution between 2012–2021, as previously described by our group (Supplementary Table 1A–C) [22]. This study was performed in accordance with the Declaration of Helsinki. All patients signed a written informed consent form approved by the Institutional Review Board. We obtained data from multiple prospective investigator-initiated clinical trials in 75% (*N* = 65) of the patients (NCT03404193, NCT03661307, NCT04140487) and 25% (*N* = 22) from patients who received the same treatment regimens outside of a clinical trial (Supplementary Table 2). Data were collected under protocols DR09-0223 and PA12-0395 for retrospective data collection in patients with *FLT3* mutated AML.

### Treatment Regimens

**Fig. 1:**
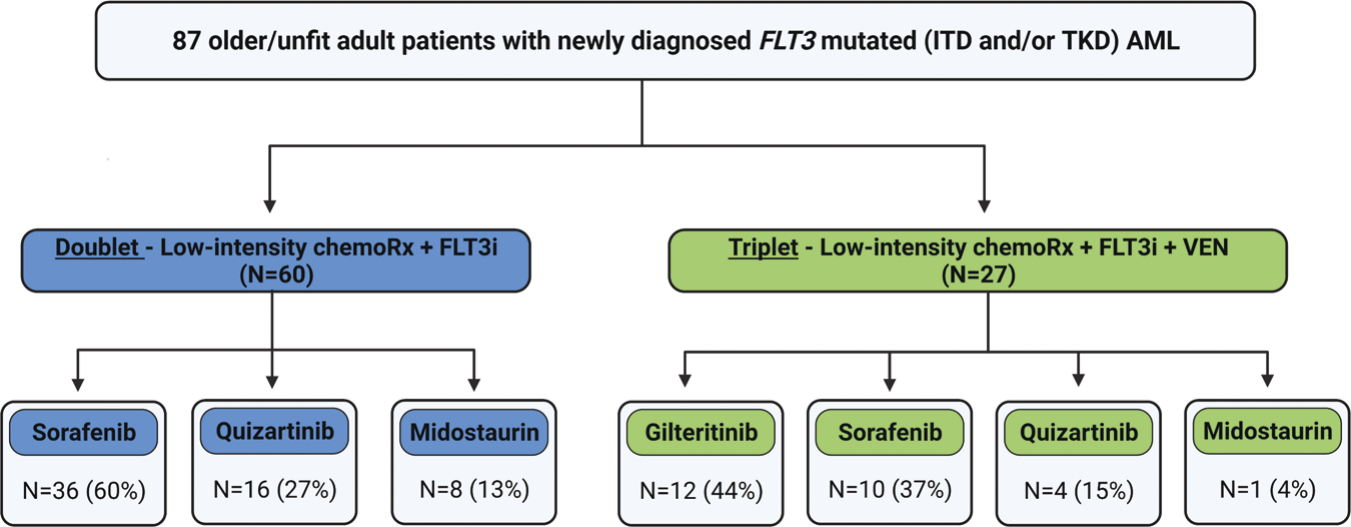
Flow diagram demonstrating specific FLT3 inhibitor used in the doublet vs. triplet arms. Among the 87 patients enrolled, 60 received doublet therapy and 27 received triplet therapy. In the doublet arm, hypomethylating agents (83%) and cladribine +/− low-dose cytarabine (17%) were used as low-intensity chemotherapy backbones. The most common FLT3 inhibitor in the doublet arm was sorafenib (60%), followed by quizartinib (27%), and midostaurin (13%). In the triplet arm, all patients received a hypomethylating agent (decitabine or azacitidine) as low-intensity chemotherapy backbone. Gilteritinib (44%) and sorafenib (37%) were the most common FLT3 inhibitors used in the triplet arm. ITD internal tandem domain, TKD tyrosine kinase domain; AML acute myeloid leukemia, FLT3i FLT3 inhibitor, VEN venetoclax.

LIC backbones consisted of decitabine, azacitidine, low-dose cytarabine (LDAC), and cladribine/LDAC in 52 (60%), 25 (29%), 7 (8%) and 3 (3%) patients, respectively (Supplementary Table 3). All patients received LIC combined with a FLT3 inhibitor (starting from day 1 of induction, given continuously). Sorafenib, quizartinib, gilteritinib, and midostaurin were used in 46 (53%), 20 (23%), 12 (14%), and 9 (10%) patients, respectively. Further treatment details regarding specific FLT3 inhibitors used in the doublet versus triplet arms are described in Fig. 1. Standard dose venetoclax (400 mg/day or dose reduced in accordance with the US label for patients on strong CYP34A inhibitors) was administered starting from day 1 to day 14 or day 28 (depending on the specific clinical trial the patient was enrolled in, or physician choice in off protocol cases) in patients who received triplet therapy, as previously described by our group[2] (see detailed treatment schema in Supplementary Fig. 1). None of the patients in the doublet arm received venetoclax during frontline therapy. At our institution, the number of *FLT3* mutated patients who received HMA with venetoclax therapy was very small as we have preferentially enrolled *FLT3* mutated patients on FLT3 inhibitor containing doublet or triplet regimens. Hence they were not included in this analysis, although the recent pooled VIALE-A and phase IB analysis provides contemporary outcome information on this population [9].

### Study design and statistics

This was a retrospective, non-randomized analysis of outcomes in *FLT3* mutated older/unfit patients treated at our institution in the specified time period. Responses were defined per international working group (IWG) 2003 criteria [23]. Composite complete remission (CRc) constituted CR and CR with incomplete blood count recovery (CRi). Response rates were recorded as the best response rates achieved at anytime point during frontline therapy. Relapse was defined by the detection of more than 5% blast in a bone marrow aspirate or by the biopsy-proven extramedullary myeloid sarcoma.

Baseline patient characteristics were evaluated using median for continuous variables and frequency for categorical variables. Categorical variables were compared for significance using the chi-square or Fisher’s exact test, and continuous variables were analyzed using the independent samples median test. OS was calculated from the start date of induction chemotherapy to the date of death due to any reason; censored at the last follow-up. Relapse-free survival (RFS) was calculated from the start date of induction therapy to the date of disease progression, death due to any cause, or last documented follow-up. Kaplan-Meier method was performed to calculate the probability of OS, and log-rank test was used to compare OS and RFS between groups of patients. Cox proportional hazards regression model was used to evaluate the effect of key variables upon OS. Statistical analyses were performed in SPSS© (version 26).

## Results

Of the 87 older/unfit patients with newly diagnosed *FLT3*-mutated AML treated at our institution, 60 (69%) and 27 (31%) received doublet (LIC + FLT3 inhibitor) and triplet (LIC + FLT3 inhibitor + Venetoclax) regimens, respectively (Fig. 1). LIC backbone consisted of HMA (83%) and cladribine plus LDAC or LDAC alone (17%) in the doublet cohort (Fig. 1). All patients (100%) in the triplet arm received HMA as the LIC backbone. Baseline clinical characteristics, including age, kidney/liver functions, cytogenetics, and molecular aberrations, were similar between the patients treated with doublet and triplet regimens (Table 1). At diagnosis, *FLT3* ITD AR was higher in the doublet arm than in the triplet arm, 0.71 versus. 0.41 (*p* < 0.01). None of the patients in the doublet arm had an isolated *FLT3*-D835 mutation, but five (18%) had an isolated *FLT3*-D835 in the triplet arm (*p* < 0.01).

**Table 1:** Clinical characteristics of patients treated with doublet vs. triplet regimens.

Characteristics	Doublet (*N* = 60) *N* (%), Median [Range]	Triplet (*N* = 27) *N* (%), Median [Range]	*P*
Median age, years	71 [51–83]	69 [40–85]	0.17
Age ≥75 years old	22 (37)	7 (26)	0.32
Male gender	30 (50)	11 (41)	0.42
Type of AML			
De novo	43 (71)	20 (74)	0.97
Secondary AML	7 (12)	3 (11)	
Therapy related	10 (17)	4 (15)	
WBC, x10^9^/L	5.3 [0.3–164]	4.2 [1–201]	0.62
Hemoglobin, g/dl	9.2 [7–13]	9.0 [6–12]	0.12
Platelets, x10^9^/L	27 [3–326]	53 [9–116]	0.01
Creatinine	0.9 [0.5–4.5]	0.9 [0.5–2.2]	0.9
Total Bilirubin	0.6 [0.2–7.9]	0.5 [0.2–1.6]	0.29
Peripheral blood blasts, %	26 [0–98]	19 [0–89]	0.7
Bone marrow blasts, %	70 [22–97]	60 [22–85]	0.39
Cytogenetics			
Diploid	37 (62)	13 (48)	0.57
Complex/−5/−7	6 (10)	5 (19)	
Other	12 (20)	7 (26)	
Insufficient Metaphase	5 (8)	2 (7)	
*FLT3* Mutation			
ITD (only)	53 (88)	21 (78)	<0.01
D835 (only)	0 (0)	5 (18)	
ITD/D835 (both)	7 (12)	1 (4)	
Baseline Allelic Ratio			
ITD	0.71 [0.06–4.1]	0.41 [0–3.34]	<0.01
D835	0 [0-0.41]	0 [0-0.46]	0.17
Other Mutations			
* NPM1*	30/58 (52)	12/27 (44)	0.53
* DNMT3A*	23/57 (40)	11/26 (42)	0.86
* RAS*	8/56 (14)	4/27 (15)	0.94
* IDH2*	8/57 (14)	5/27 (18)	0.59
* RUNX1*	4/39 (10)	3/27 (11)	0.91
* CEBPA*	5/53 (9)	1/27 (4)	0.35
* ASXL1*	3/39 (8)	2/27 (7)	0.96
* IDH1*	4/57 (7)	3/27 (11)	0.52
* TP53*	4/52 (7)	1 /27 (4)	0.49

Doublet, low-intensity chemotherapy + FLT3 inhibitor; Triplet, low-intensity chemotherapy + FLT3 inhibitor + venetoclax; *N* number, *AML* acute myeloid leukemia, WBC white blood cell, L liter, dl deciliter, ITD internal tandem duplication.

Prior to the availability of gilteritinib in 2018, sorafenib and HMA combination was the most commonly used therapy for older/unfit patients at our institution. Hence, sorafenib was the most common FLT3 inhibitor of choice (60%) for patients who received doublet therapy (mostly between 2012-2017) until we started exploring triplet regimens in 2018 onwards once HMA with venetoclax was US FDA approved (Fig. 1). With the FDA approval of gilteritinib in 2018 and the more recent time frame of use of triplets, gilteritinib was the most common FLT3 inhibitor (44%) in patients who received triplet therapy (Fig. 1).

### Response to Therapy

**Fig. 2:**
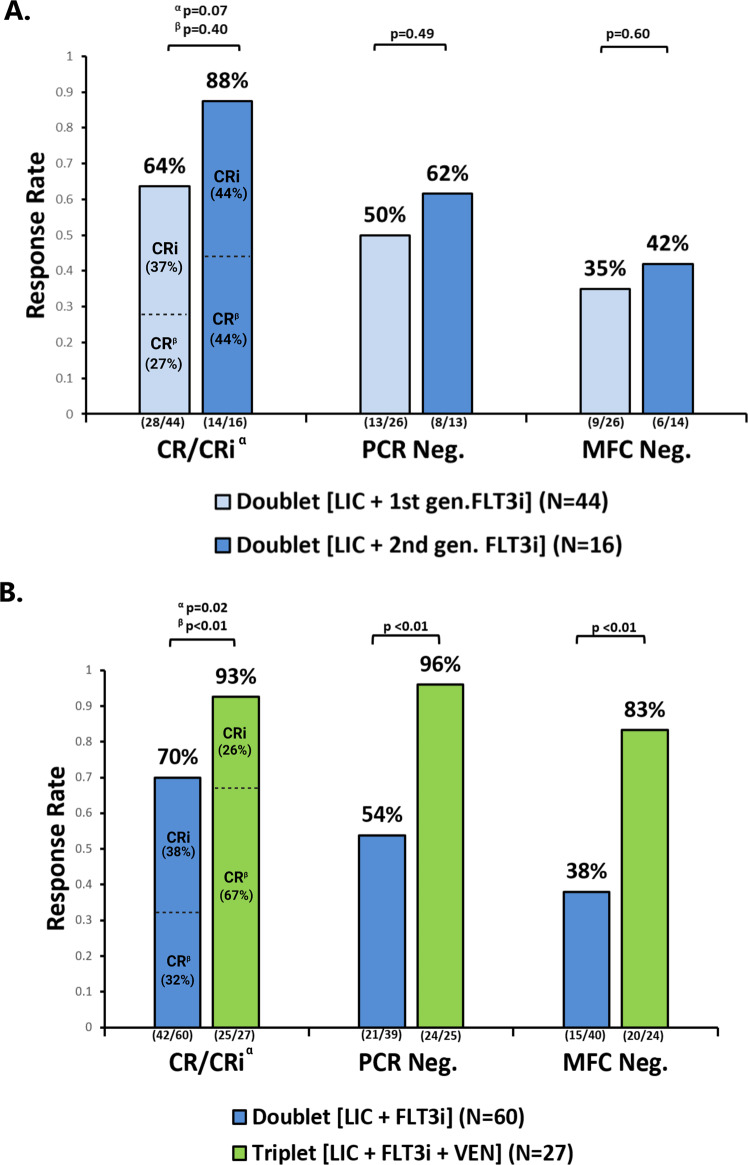
Comparison of remission rates and measurable residual disease (MRD) negativity rates in patients treated with 1st generation FLT3i doublets vs. 2nd generation FLT3i doublets (A) and doublets vs. triplets (B). **A**. Of the 60 patients treated with the doublet regimens, 44 (73%) received a first-generation FLT3i and 16 (27%) a second-generation FLT3i. We noted no statistically significant difference in CR/CRi (64% versus 88%, *P* = 0.07^α^),CR (27% versus 44%, *P* = 0.40^β^), FLT3 PCR (50% versus 62%, *P* = 0.49) or MFC negativity (35% versus 42%, *P* = 0.60) rates in patients treated with first- (*n* = 44) or second-generation (*n* = 16) FLT3i based doublets. Both PCR and MFC testing were missing in 2 responders in group treated with a 1^st^ generation FLT3 inhibitor. PCR and MFC testing were missing in 3 and 2 responders, respectively, in group received a 2^nd^ generation FLT3 inhibitor. **B** The rates of CR/CRi (93 versus 70%, *P* = 0.02^α^), FLT3 PCR negativity (96% versus 54%, *P* < 0.01), and MFC negativity (83% versus 38%, *P* < 0.01) were significantly higher with the triplet regimen than with the doublet regimen. Triplet regimens not only resulted in higher CR/CRi rates, but importantly in a meaningfully higher true CR rates (67% versus 32%, *P* < 0.01^β^). PCR and MFC testing were missing in 3 and 2 responders, respectively in doublet arm. MFC testing was missing in 1 responder in triplet arm. MRD assessments were performed by multicolor flow cytometry (MFC, sensitivity of 10^−3^) and multiplex polymerase chain reaction (FLT3-PCR, sensitivity of 10^−2^) for ITD and kinase domain (D835). LIC low intensity chemotherapy, FLT3i FLT3 inhibitor, VEN venetoclax; CR/CRi complete response/complete response with incomplete count recovery, PCR RT-polymerase chain reaction assay for FLT3; MFC multicolor flow cytometry; Neg., negative.

Of the 60 patients treated with the doublet regimens, 44 (73%) received a first-generation FLT3 inhibitor (36 sorafenib, 8 midostaurin) and 16 (27%) a second-generation FLT3 inhibitor (quizartinib). We noted no statistically significant difference in CR/CRi (64% versus 88%, *P* = 0.07), CR (27% versus 44%, *P* = 0.40), FLT3-PCR negativity (50% versus 62%, *P* = 0.49) or MFC negativity (35% versus 42%, *P* = 0.60) rates in patients treated with first- (*n* = 44) or second-generation (*n* = 16) FLT3 inhibitor based doublets **(**Fig. 2A**)**.

In the triplet group, 12 (44%), 10 (37%), 4 (15%), and 1 (4%) patients received gilteritinib, sorafenib, quizartinib, and midostaurin added to LIC and venetoclax backbone, respectively. Triplet therapy was associated with significantly higher CR/CRi (93% versus 70%, *P* = 0.02), FLT3-PCR negativity (96% versus 54%, *P* < 0.01), and MFC negativity (83% versus 38%, *P* < 0.01) rates compared with the doublet regimens (Fig. 2B). Triplet regimens not only resulted in higher CR/CRi rates, but importantly in a meaningfully higher true CR rates (67% versus 32%, *P* = 0.002).

**Fig. 3:**
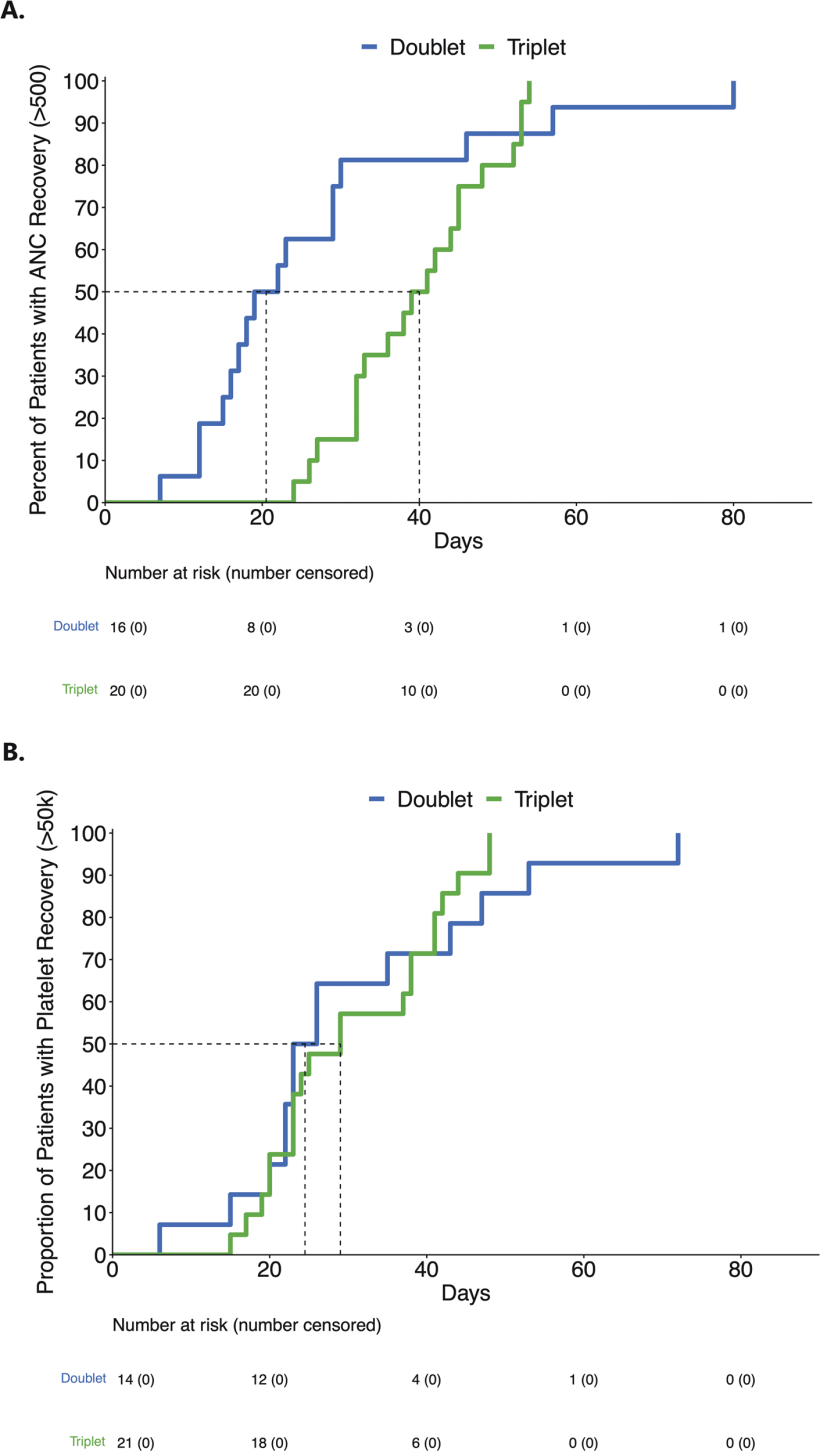
Time to neutrophil (A) and platelet recovery (B) at the end of cycle 1 with doublet vs. triplet regimens. **A** *T*he median time to absolute neutrophil count (ANC) > 500/mm^3^ is 21 days (95% CI: 16–46 days) in the doublet group and 40 days (95% CI: 33–48 days) in the triplet group, HR 1.68 (95% CI: 0.83–3.41), *p* = 0.15. **B** The median time to platelet > 50,000 microliter is 25 days (95% CI: 22–53 days) in the doublet group and 29 days (95% CI: 23–41 days) in the triplet group. HR 0.84 (95% CI: 0.41–1.72), *p* = 0.6. ANC absolute neutrophil count (per mm^3^); Platelet (per microliter); 50k, 50,000.

Responses occurred earlier with the triplet. The median cycles to achieve the best response were 1 [range 1-4] and 2 cycles [range 1–5] with triplet and doublet regimens, respectively. At the end of cycle 1, the CR/CRi (85% [*N* = 23] versus. 43% [*N* = 26], *P* < 0.01) and CR (48% [*N* = 13] versus 12% [*N* = 7], *P* < 0.01) rates were significantly higher in the triplet versus doublet group (Supplementary Table 4). Responses beyond cycle 1 were more common with the doublet: 8% and 27% of the patients achieved CR/CRi in cycle #2 or later in triplet and doublet arms, respectively. The median days to ANC recovery >500/mm^3^ among patients who achieved a CRc on triplet versus doublet regimens was 40 versus 21 days (*P* = 0.15). The median days to platelet recovery >50,000/mcL among patients who achieved a CRc on triplet versus doublet regimens was 29 versus 25 days, *P* = 0.6 (Fig. 3). Interestingly, when all patients were considered, 14/27 (52%) versus 20/60 (33%) (*P* = 0.1) of triplet versus doublet patients had an absolute ANC > 500/mm3 by Day 42 from the start of therapy (Day 1). By Day 42 of therapy, 20/27 (74%) versus 17/60 (28%) (*P* < 0.01) of patients on triplet versus doublet had a platelet count greater than 50,000/mcL. This was reflected in the similarity of 60-day mortality between triplets and doublets; 7% (*n* = 2) versus 10% (*n* = 6), *P* = 0.70, respectively.

Deaths in CRc were noted in 3 (11%) patients treated on triplets and were attributed to infections (*N* = 2), and unknown (*N* = 1). Deaths in CRc were noted in 12 (20%) patients treated on doublets and were attributed to infection (*N* = 5), unknown (*N* = 5), post-ASCT complication (*N* = 1), intracranial hemorrhage (*N* = 1). One (4%) and 4 (6%) patients discontinued therapy due to treatment-related adverse events on triplet versus doublet regimens.

### Survival

**Fig. 4:**
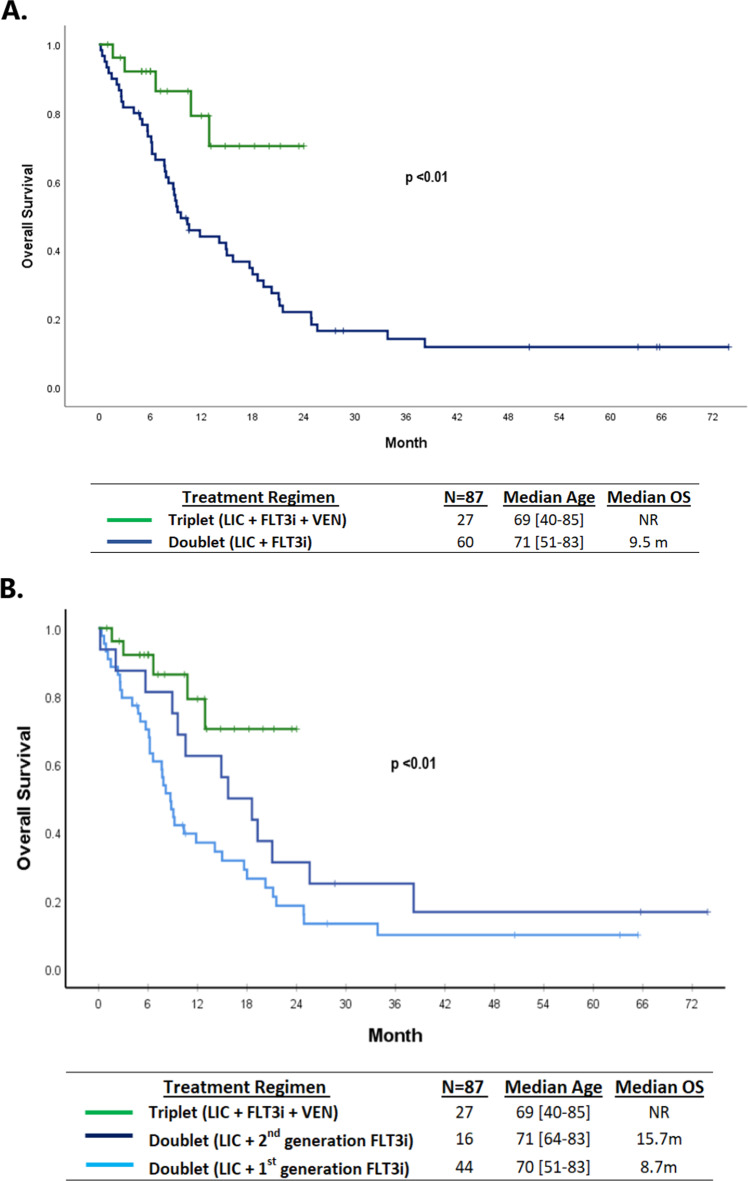
Overall survival in patients treated with doublets vs. triplets (**A**) and triplets vs. 1^st^ generation FLT3i-based doublets vs. 2^nd^ generation FLT3i-based doublets (**B**). **A** The median follow-up time was shorter in the triplet arm than in the doublet arm, 12 versus 63 months (*p* < 0.01), reflecting the more recent use of triplet regimens at our institution (mostly mid-2018 onwards). Patients treated with a triplet regimen (green) achieved longer median OS compared with patients treated with a doublet regimen (dark blue) (NR versus 9.5 months, *P* < 0.01). **B** The median OS in patients treated in the three groups: Triplets (green), doublets with second-generation (quizartinib or gilteritinib) FLT3 inhibitors (dark blue), and doublets with first-generation (midostaurin or sorafenib) FLT3 inhibitors (light blue) were NR, 15.7 months, and 8.7 months, respectively (*P* < 0.01). There was no statistically significant OS difference between patients treated with first- vs. second-generation FLT3i-based doublets (*P* = 0.19). LIC low intensity chemotherapy, FLT3i FLT3 inhibitor, VEN venetoclax, OS overall survival, m month, NR not reached.

The median follow-up time was shorter in the triplet arm than in the doublet arm, 12 versus 63 months (*p* < 0.01), reflecting the more recent use of triplet regimens at our institution (mostly mid-2018 onwards). Patients treated with a triplet regimen achieved longer median OS compared with patients treated with a doublet regimen (NR versus 9.5 months, *P* < 0.01) (Fig. 4A). The median OS in patients treated in the three groups: Triplets, doublets with second-generation (quizartinib or gilteritinib) FLT3 inhibitors, and doublets with first-generation (midostaurin or sorafenib) FLT3 inhibitors, were NR, 15.7 months, and 8.7 months, respectively (*P* < 0.01) (Fig. 4B). There was no statistically significant OS difference between patients treated with first- versus second-generation FLT3 inhibitor based doublets (*P* = 0.19) (Fig. 4B). RFS was also longer in the triplet arm than in the doublet arm (*p* = 0.03) (Supplementary Fig. 2).

In the triplet arm, 11 patients (40%) received a first-generation FLT3 inhibitor (10 sorafenib and 1 midostaurin) and 16 (60%) received a second-generation FLT3 inhibitor (12 gilteritinib and 4 quizartinib) added to the HMA and venetoclax backbone. Although the OS numerically appeared to be the best in patients treated with a second-generation FLT3 inhibitor based triplet regimen (Supplementary Fig. 3A, B), this was not statistically significant with median OS currently NR for both the first- and second-generation FLT3 inhibitor triplets (*P* = 0.37), possibly due to the small comparator numbers in these two subsets.

We performed a subgroup analysis to determine the effect of FLT3-ITD AR (high [≥0.5] vs. low [<0.5]) on OS. In comparison to doublet regimens, triplet regimens were associated with a significantly longer OS in patients with a high AR (not reached vs. 9.1 months) or a low AR (not reached vs. 15.7 months) (Supplementary Fig. 4A, B**)**. This finding, however, did not reach statistical significance due to the small sample size. Additionally, we used cox regression analysis to determine the effect of AR and treatment regimen on OS and discovered that AR (high vs. low) had no significant effect on OS (HR 0.7, CI [0.4–1.3], *p* = 0.28) when evaluated in the same model. On the other hand, treatment with a doublet regimen was associated with inferior OS (HR 3.2, CI [1.2–9.2], *p* = 0.024). (Supplementary Table 5)

### Measurable residual disease

The median time to the best MFC-based MRD (from the date of treatment start to the date that best MRD response achieved) was 2.6 m (range, 0.6–9.0 m). Achievement of MFC negativity (sensitivity of 10^−3^) was associated with superior OS. The median OS was 21 months in MFC negative patients versus 14.8 months in patients with positive MFC as best MRD response, *P* = 0.02 **(**Supplementary Fig. 5A**)**. However, FLT3-PCR negativity was not associated with statistically significant OS improvement **(**Supplementary Fig. 5B**)**, possibly due to the lower sensitivity (sensitivity of 10^−2^) of our in-house FLT3-PCR assay used in these analyses. The higher MFC negativity (83% versus 38%, *P* < 0.01) rates with the triplet may also have contributed to the earlier and better restoration of hematopoiesis, resulting in the higher true CR rates and improved OS.

### Allogeneic stem cell transplantation

With a median time to ASCT of 4 months [range 2.5–7.6 months], 14 patients (16%) underwent ASCT in CR1. A landmark analysis **(**Supplementary Fig. 6A**)** at 4-month (median time to ASCT) (*n* = 50) demonstrated that patients who received ASCT in CR1 had superior OS than patients who did not receive ASCT in CR1; the median OS was NR and 19 months, respectively, *p* = 0.01 **(**Supplementary Fig. 6B**)**. Patients treated with triplet regimens were more likely to proceed to ASCT in CR1 than patients treated with doublet regimens potentially due to the higher CR/CRi and CR rates with the triplet; 30% (*N* = 8) vs. 10% (*N* = 6), respectively (*P* = 0.02). Using the same 4-month landmark analysis, we separately analyzed patients treated with a doublet and triplet regimen. We observed that ASCT appeared to impact OS in patients treated with doublets positively; the median OS was NR versus 18.5 months in ASCT versus no ASCT patients, *p* = 0.016 (Supplementary Fig. 6C). However, the median OS in the triplet group was similar irrespective of ASCT; Not reached in both transplanted and non-transplanted patients (*P* = 0.82) (Supplementary Fig. 6D). Of note, this analysis is quite limited by the triplet arm’s limited numbers and short follow-up. Among ASCT recipients, 3 patients died postASCT: 1 patient was treated with azacitidine + venetoclax + sorafenib, and relapsed at post-ASCT day 90; 1 patient was on LDAC + quizartinib and relapsed at post-ASCT day 100; 1 patient received azacitidine + sorafenib, and died in CR 2 years after ASCT due to anticoagulant related major bleeding.

## Discussion

First- and second-generation FLT3 inhibitor based doublet regimens were associated with comparable CRc and CR rates and OS in older/unfit adults with newly diagnosed *FLT3* mutated AML. However, the addition of venetoclax to the HMA and FLT3 inhibitor combination was associated with improved outcome parameters in this retrospective, contemporary analysis of frontline older/unfit FLT3 mutated patients treated at our institution. The CR/CRi, CR rates, MFC negativity, and the 2-year OS rates were all statistically and clinically meaningfully improved at 93% vs. 70%, 67% vs. 32%, 83% vs. 38%, and 70% vs. 22% in patients treated with frontline triplet and doublet regimens, respectively (Figs. 2B and 4A). The 60-day mortality rates (7% [*n* = 2] vs 10% [*n* = 6]) and death in CRc rates (11% [*n* = 3] vs 20% [*n* = 12]), were similar between the triplet and doublet regimens, respectively, potentially due to a more than doubling of the CR rates with triplets which surprisingly led to a higher absolute proportion of patients achieving ANC > 500/mm^3^ (52% versus 33%) and platelets count >50,000/mcL (74% versus 28%) by Day 42 from the start of the triplet versus the doublet therapy. These data suggest that triplets could be safely delivered in this frontline older/unfit population with optimal monitoring and dose adjustments in centers with experience with venetoclax based regimens and adequate supportive care capabilities.

Patients receiving the triplet regimen achieved their best response more rapidly than patients receiving the doublet regimen. In patients who received triplet or doublet regimens, the median number of cycles to achieve CR/CRi was 1 and 2, respectively. However, among the group of patients who responded at the end of the first cycle, it is important to note that the time to achieve ANC > 500 was indeed longer with triplet regimens (median 40 days) than with doublet regimens (median 21 days). These findings suggest that, while recovering counts takes longer, with triplet regimen, patients are more likely to achieve their best response faster, and eventually, more of the patients will have a proper count recovery but patients need to be closely followed and supported through the initial cycles of the triplet with an expectation of antecedent myelosuppression. It is important to note that the majority of the patients reported in this study were treated at our institution in prospective clinical trials (Supplementary table 2**)**. In earlier phases of these trials, in cycle 1, patients in the triplet cohort were given venetoclax and FLT3 inhibitors for up to 28 days (Supplementary Fig. 1). Later, due to the prolonged count recoveries observed, these protocols were amended to limit the duration of venetoclax and FLT3 inhibitors to 14 days or less with incorporation of a Cycle 1 Day 14 bone marrow. With these modifications, ANC recovery time was reduced to less than 40 days. This reduction in the duration of the venetoclax to 14 days in cycle 1 and if needed further reduction in the venetoclax duration to allow for improved count recovery in subsequent cycles is a key point to highlight as this approach is different from what is frequently done with the HMA-venetoclax doublet wherein 21–28 days of venetoclax are often given in cycle 1 of therapy. These phase I/II clinical trials (NCT03404193, NCT03661307, NCT04140487) continue to enroll patients to optimize triplet dose schedule in *FLT3* mutated AML.

Treatment options and outcomes for older adults with *FLT3* mutated AML who are unsuitable for intensive induction chemotherapy are limited, and improvements are urgently needed [4, 5]. The expected median OS with HMA with FLT3 inhibitors (HMA with sorafenib, HMA with midostaurin, HMA with gilteritinib) or HMA with venetoclax induction regimen are 8 to 12.5 months, with 2-year OS of <30% with any of these doublets [4, 5, 7, 9]. Similarly, in our analysis, patients treated with first-generation FLT3 inhibitor based doublet regimens (with sorafenib or midostaurin) had a median OS of 8–9 months. Although statistically not significant, likely due to the small comparator numbers (N of 16), the second-generation FLT3 inhibitor based doublets (all with quizartinib) in our analysis resulted in a numerically longer median OS (16 months) than first-generation doublets (Fig. 4B). A total of 27 patients were treated with triplet regimens, which included HMA, a FLT3 inhibitor, and venetoclax. The median OS was not reached in patients treated with triplet regimens with a projected 2-year OS of 70%, although it must be noted that the median follow-up for the triplets at this time is only 12 months as the triplet approach (on or off clinical trials) is relatively new starting around mid-2018 when HMA with venetoclax was US FDA approved and emerged as the frontline standard of care for the older/unfit AML patients (Fig. 4A).

While the observed outcomes with triplet therapies were statistically superior to those with doublet regimens in this retrospective single center analysis, this study has limitations and should be interpreted accordingly. The median duration of follow-up was significantly shorter in the triplet arm (12 vs. 63 months, p0.01). Events that occurred later in the course of treatment may have been overlooked and continued follow-up for more mature OS will be needed. Although the vast majority of patients received HMA as a backbone therapy, 17% of the doublet arm received LDAC-based regimens which may introduce bias in the comparisons. It is also important to note that the triplet arm had nearly half the sample size of the doublet arm. Furthermore, although the triplet appeared safe with early mortality <10% in spite of prolonged neutropenia this was in the setting of a tertiary cancer center with extensive experience with venetoclax based regimens, dose interruptions in the setting of prolonged neutropenia, curtailed venetoclax duration in subsequent cycles to avoid cumulative myelosuppression, the use of prophylactic antimicrobials, and careful laboratory monitoring and close follow-up. An awareness of the potential myelosuppression of the regimen and close monitoring and interventions to mitigate myelosuppression will be needed to successfully implement this regimen on a broader sale. Eventually carefully designed phase II and phase III studies are needed to confirm the efficacy and safety, and to resolve the limitations noted in this retrospective analysis.

In conclusion, first- and second-generation FLT3 inhibitor based doublet regimens were associated with comparable CR/CRi rates and median OS of 9–16 months in older/unfit adults with newly diagnosed *FLT3* mutated AML. The triplet regimens (HMA + FLT3 inhibitor + venetoclax) significantly improved CR/CRi rates, CR rates, MRD negativity rates, and OS leveraging the synergy of venetoclax and FLT3 inhibitor, without increasing 60-day mortality or deaths in remission in this retrospective single-center analysis. As seen with many venetoclax combinations, the median time to ANC and platelet recovery was increased in responders to the triplet versus the doublet, especially prominent during cycle 1 and awareness of this early prolonged myelosuppression is important. Surprisingly however the absolute number of patients who had ANC > 500/mm^3^ and platelets count >50,000/mcL by Day 42 from the start of therapy was higher with the triplet, likely due to the more than doubling of CR rate with the triplet. Moving forward the duration of venetoclax in the first and subsequent cycles, the dose of the individual FLT3 inhibitors used in the triplets, the timing of bone marrow, duration of triplet therapy before shifting to some form of maintenance and transportability and safety of this regimen outside of academic centers with expertise in treating AML must be evaluated in larger prospective (and ideally randomized) frontline studies to confirm the efficacy and safety signals noted in our analysis before such therapy can be safely, widely adopted, especially outside of larger and more experiences academic centers.

## Supplementary information

Supplemental Tables and Figures
