# Supplementary material for: Hypomethylating agent and venetoclax with FLT3 inhibitor “triplet” therapy in older/unfit patients with FLT3 mutated AML
Source: Blood Cancer J. 2022 May 2;12(5):77. doi: 10.1038/s41408-022-00670-0 (PMC9061716; doi:10.1038/s41408-022-00670-0)

**Supplement Table 1: Molecular Assessments (Alotaibi, Yilmaz et al, 2021, Blood Cancer Discovery)**

A multiplex polymerase chain reaction (PCR) analysis for internal tandem duplications (ITD) and kinase domain (D835) mutations in FLT3 was performed on DNA isolated from bone marrow aspirate samples as previously described by our group {Luthra, 2014, Next-generation sequencing-based multigene mutational screening for acute myeloid leukemia using MiSeq: applicability for diagnostics and disease monitoring}. Briefly, fluorescently-labeled PCR primers were utilized to amplify targeted juxtamembrane domain and kinase domain sequences. PCR product sizes were determined using capillary gel electrophoresis on a 3100 genetic analyzer (Applied Biosystems, Foster City, CA). The presence of PCR fragment larger than the wild-type allele was considered to constitute ITD. For D835 mutations, PCR products were digested with the Eco RV restriction enzyme prior to capillary electrophoresis. The wild-type allele cut by this enzyme result in 2 fragments, whereas mutations at D835 alter the Eco RV recognition site and result in one fragment. The allelic frequency (AF) is determined as the area under the curve of the mutant allele divided by the sum of mutant and wild-type alleles. The analytical sensitivity of this assay is approximately 1% mutant allele in a background of wild-type allele (detailed methodology reported by Luthra, 2014, Next-generation sequencing-based multigene mutational screening for acute myeloid leukemia using MiSeq: applicability for diagnostics and disease monitoring}.

NGS was performed on clinical-grade CLIA-compliant myeloid NGS Illumina MiSeq (Illumina, Inc., San Diego, CA, USA) platforms developed at MDACC, to evaluate the entire coding sequences of 28, 53 or 81 AML-related genes (detailed in this table). A minimum of 5 ng of DNA were required for targeted sequencing. The assays survey for mutations in hotspot genomic loci (such as EZH2, DNMT3A, GNAS, IDH1, IDH2, JAK2, KIT, KMT2A [MLL], MPL, NPM1, NOTCH1, NRAS, KRAS, and TP53) or exonic regions (such as ABL1, EGFR, GATA2, IKZF2, MDM2, NOTCH1, RUNX1, ASXL1, EZH2, HRAS, JAK2, KMT2A, NPM1, TET2, BRAF, IDH1, KIT, NRAS, TP53, DNMT3A, GATA1, IDH2, KRAS, MYD88, PTPN11, WT1). In addition, FLT3 (internal tandem duplication (ITD) and D835) were mutations are assessed by polymerase chain reaction followed by capillary electrophoresis on the Genetic Analyzer platform (Applied Biosystems, Foster City, CA, USA), as described in the previous paragraph.{Warren, 2012, Clinical impact of change of FLT3 mutation status in acute myeloid leukemia patients} Based on validation, a minimum of 5% variant allele frequency as a detection limit and a minimum depth of coverage of 250 reads were set as cut-off.

**Table 1A. 81 gene panel**

**Gene Exons (codons) tested**

ANKRD26 (NM_014915) 1 (1-6)

ASXL1 (NM_015338) 11-12 (362-1442), 12 (1450-1542)

ASXL2 (NM_018263) 11-12 (381-1436)

BCOR (NM_017745) 2-4 (1-511), 4-15 (515-1644), 15 (1663-1722)

BCORL1 (NM_021946) 1-6 (1-1261), 6 (1292-1323), 6-12 (1326-1700), 12 (1706-1712)

BRAF (NM_004333) 11 (439-478), 15 (581-620)

BRINP3 (NM_199051) 2-8 (1-767)

CALR (NM_004343) 9 (352-418)

CBL (NM_005188) 7-9 (336-477)

CBLB (NM_170662) 7-9 (282-397), 10 (402-469)

CBLC (NM_012116) 7-9 (336-454), 10 (465-475)

CEBPA (NM_004364) 1 (1-96), 1 (249-358), 1 (215-244), 1 (128-175), 1 (178-201)

CREBBP (NM_004380) 1-8 (1-608), 9-31 (615-1943), 31 (1950-2443)

CSF3R (NM_156039) 14 (575-622), 17 (681-864)

CUX1 (NM_181552) 2-6 (11-172), 6-9 (174-241), 10-14 (248-408)

DDX41 (NM_016222) 1-17 (1-623)

DNMT3A (NM_022552) 8-22 (286-862), 23 (866-913)

EED (NM_003797) 1-2 (1-69), 2-8 (71-287), 9-12 (289-442)

ELANE (NM_001972) 1-2 (1-48), 2 (69-75), 3-5 (102-268)

ETNK1 (NM_018638) 3 (228-275)

ETV6 (NM_001987) 1-8 (1-453)

EZH2 (NM_004456) 2-5 (1-158), 5-6 (160-205), 7 (209-217), 8-19 (243-732), 20 (752)

FBXW7 (NM_033632) 9-12 (413-708)

FLT3 (NM_004119) 11-20 (437-847)

GATA1 (NM_002049) 2-3 (1-84)

GATA2 (NM_032638) 2-5 (1-377), 5-6 (379-481)

GFI1 (NM_005263) 2 (2-39)

GNAS (NM_000516) 8 (200-202), 11 (315-324)

HNRNPK (NM_002140) 3-17 (1-465)

HRAS (NM_005343) 2-3 (1-70), 3-4 (74-150)

IDH1 (NM_005896) 4 (132-133)

IDH2 (NM_002168) 4 (125-178)

IKZF1 (NM_006060) 2-8 (1-443), 8 (445-518)

IL2RG (NM_000206) 1-8 (1-370)

IL7R (NM_002185) 5-7 (180-292)

JAK1 (NM_002227) 3-22 (3-1023), 22-24 (1026-1123)

JAK2 (NM_004972) 10 (405-442), 12-14 (505-622), 16 (665-711), 18 (762-803)

JAK3 (NM_000215) 2-23 (1-1069)

KDM6A (NM_021140) 1-19 (1-971), 19-21 (980-1070), 22-29 (1080-1402)

KIT (NM_000222) 8-9 (411-514), 11 (550-592), 17 (788-828)

KMT2A (NM_005933) 2 (145-168), 3-4 (176-1075), 4 (1081-1112), 5 (1117-1184), 6 (1190-1212), 7 (1224-1325), 8-10 (1338-1440), 11-13 (1445-1560), 14-15 (1566-1665), 27 (2186-2195), 27 (2201-2355), 27 (2373-3215), 27 (3223-3324), 27 (3339-3575)

KRAS (NM_004985) 2-4 (1-150)

MAP2K1 (NM_002755) 2 (27-90), 3 (98-146)

MPL (NM_005373) 10 (490-522), 12 (552-636)

NF1 (NM_001042492) 2-5 (21-189), 6 (201-218), 8-13 (244-467), 13-17 (478-667), 18 (674-728), 18-22 (746-992), 23-24 (997-1066), 25-26 (1082-1146), 26-30 (1160-1370), 31-35 (1382-1550), 35-38 (1564-1868), 39 (1870-1884), 39-47 (1886-2322), 47-52 (2325-2555), 52-58 (2568-2840)

NOTCH1 (NM_017617) 26-28 (1529-1795), 34 (2061-2286), 34 (2290-2556), 34 (2061-2286), 34 (2290-2556)

NPM1 (NM_002520) 11 (283-295)

NRAS (NM_002524) 2-4 (1-150)

PAX5 (NM_016734) 1-10 (8-392)

PHF6 (NM_032458) 2-3 (1-78), 4-10 (81-366)

PIGA (NM_002641) 2 (1-6), 2-6 (16-485)

PML (NM_033238) 3 (201-255)

PRPF40B (NM_001031698) 2-19 (2-609), 19-20 (611-658), 20-26 (661-893)

PTEN (NM_000314) 7-8 (212-285), 8 (310-339)

PTPN11 (NM_002834) 3-4 (46-125), 7 (253-285), 12 (460-462), 12-13 (465-533)

RAD21 (NM_006265) 2-3 (1-82), 4-14 (92-632)

RARA (NM_000964) 6-7 (211-338)

RUNX1 (NM_001754) 2-9 (1-438), 9 (456-474)

SETBP1 (NM_015559) 4 (838-885)

SF1 (NM_004630) 1-13 (1-640)

SF3A1 (NM_005877) 1-9 (1-424), 9-16 (427-794)

SF3B1 (NM_012433) 13-16 (574-790)

SH2B3 (NM_005475) 2 (1-121), 2 (129-170), 2 (189-205), 2-8 (211-576)

SMC1A (NM_006306) 1-25 (1-1234)

SMC3 (NM_005445) 1 (1-5), 2-6 (19-110), 6-16 (113-504), 16-17 (507-580), 17-25 (591-975), 25-27 (979-1151), 28-29 (1159-1217)

SRSF2 (NM_003016) 1 (1-38), 1 (45-121)

STAG1 (NM_005862) 2 (1-5), 3-12 (10-392), 13-20 (402-703), 21-22 (718-738), 22-27 (740-953), 27-34 (955-1259)

STAG2 (NM_006603) 2-15 (1-512), 16-20 (521-699), 21-33 (714-1232)

STAT3 (NM_139276) 17-22 (489-715)

STAT5A (NM_003152) 3-7 (1-214), 8-9 (249-286), 9-20 (303-795)

STAT5B (NM_012448) 16 (636-693)

SUZ12 (NM_015355) 1-2 (17-107), 4-5 (129-169), 7-16 (198-740)

TERT (NM_198253) 1 (1-24), 1-2 (33-172), 2-4 (246-630), 4-16 (633-1133)

TET2 (NM_001127208) 3 (1-77), 3 (91-826), 3 (829-853), 3-11 (867-2003)

TP53 (NM_000546) 2 (1-25), 4-11 (80-394)

U2AF1 (NM_006758) 2 (15-44), 6 (117-161)

U2AF2 (NM_007279) 1-5 (1-161), 6-12 (163-473)

WT1 (NM_024426) 1 (122-216), 1 (2-59), 1 (70-104), 2-10 (216-518)

ZRSR2 (NM_005089) 1-4 (1-90), 5 (108-131), 6-9 (134-263), 9-11 (268-483)

Note. *CRLF2* (NM_022148.2) exon 6 (codons 217-256) is also targeted in this assay, and coverage for this area has been manually reviewed to be adequate. **Coverage by gene and codon(s) tested with >250x coverage.**

**Table 1B. 53 gene panel**

**Gene Exons (codons) tested**

ABL1 (NM_005157) 4-6 (243-362), 7 (395-424)

AKT1 (NM_005163) 3 (16-49)

ALK (NM_004304) 23 (1172-1175), 25 (1248-1275)

APC (NM_000038) 16 (875-918), 16 (1113-1153), 16 (1257-1575)

ATM (NM_000051) 8 (353-355), 9 (409-412), 12 (601-633), 17 (846-880), 26 (1308-1331), 34 (1678-1719), 35 (1741-1773), 36 (1792-1832), 39 (1940-1973), 50 (2441-2479), 54 (2665-2670), 55 (2694-2717), 56 (2725-2756), 59 (2889-2891), 61 (2946-2950), 63 (3007-3051)

BRAF (NM_004333) 11 (439-471), 15 (581-606)

CDH1 (NM_004360) 3 (77-117), 8 (369-379), 9 (399-439)

CDKN2A (NM_000077) 2 (51-70)

CSF1R (NM_005211) 7 (297-301), 22 (926-970)

CTNNB1 (NM_001904) 3 (12-50)

DNMT3A (NM_022552) 23 (866-913)

EGFR (NM_005228) 3 (108-142), 7 (288-297), 15 (598-627), 18-20 (708-817), 21 (857-875)

ERBB2 (NM_004448) 19 (754-769), 20 (772-818), 21 (839-883)

ERBB4 (NM_005235) 3 (98-140), 4 (153-186), 6 (208-244), 7 (248-287), 8 (295-306), 9 (333-350), 15 (579-619), 23 (907-936)

EZH2 (NM_004456) 16 (618-649)

FBXW7 (NM_033632) 5 (243-278), 8 (375-394), 9 (429-471), 10 (473-508), 11 (549-583)

FGFR1 (NM_015850) 4 (120-126), 7 (247-250)

FGFR2 (NM_000141) 7 (250-313), 9 (362-382), 12 (521-550)

FGFR3 (NM_000142) 7 (247-288), 9 (379-422), 14-15 (639-659), 18 (792-807)

FLT3 (NM_004119) 11 (437-456), 14 (569-605), 16 (648-683), 20 (807-843)

GNA11 (NM_002067) 4-5 (159-216), 6-7 (255-360)

GNAQ (NM_002072) 4-7 (159-360)

GNAS (NM_000516) 8 (200-220)

HNF1A (NM_000545) 3 (205-238), 4 (271-314)

HRAS (NM_005343) 2 (1-15), 3 (38-63)

IDH1 (NM_005896) 4 (90-132)

IDH2 (NM_002168) 4 (125-178)

JAK2 (NM_004972) 14 (615-622)

JAK3 (NM_000215) 13 (568-573), 16 (683-723)

KDR (NM_002253) 6 (220-248), 7 (267-276), 11 (471-476), 19 (872-874), 21 (946-985), 26 (1135-1146), 27 (1171-1211), 30 (1308-1357)

KIT (NM_000222) 2 (51-93), 9-11 (502-592), 13 (641-664), 14 (670-712), 15 (714-745), 17 (815-828), 18 (838-866)

KLHL6 (NM_130446) 1 (1-98)

KRAS (NM_004985) 2 (1-22), 3 (38-63), 4 (103-147)

MET (NM_001127500) 2 (168-209), 2 (375-400), 14 (1008-1028), 16 (1110-1132), 19 (1247-1284)

MLH1 (NM_000249) 12 (383-426)

MPL (NM_005373) 10 (514-522)

NOTCH1 (NM_017617) 26 (1562-1601), 27 (1673-1679)

NPM1 (NM_002520) 11 (283-295)

NRAS (NM_002524) 2 (1-18), 3 (38-62)

PDGFRA (NM_006206) 12 (552-592), 14 (659-668), 15 (673-717), 18 (823-854)

PIK3CA (NM_006218) 2 (83-118), 5 (345-353), 8 (418-445), 10 (538-555), 14 (701-729), 21 (988-1069)

PTEN (NM_000314) 1 (5-27), 3 (67-70), 6 (170-210), 7 (212-266), 8 (287-342)

PTPN11 (NM_002834) 3 (59-104), 13 (501-533)

RB1 (NM_000321) 4 (127-158), 6 (199-203), 11 (357-376), 18 (570-605), 20 (659-700), 21 (703-733), 22 (746-775)

RET (NM_020975) 10-11 (610-667), 13 (766-798), 15 (880-910), 16 (918-934)

SMAD4 (NM_005359) 3 (119-142), 5 (167-208), 6 (243-263), 8 (310-319), 9 (329-373), 10 (385-424), 11 (443-480), 12 (496-535)

SMARCB1 (NM_003073) 2 (39-78), 4 (156-167), 5 (199-210), 9 (381-386)

SMO (NM_005631) 3 (197-242), 5 (323-366), 6 (403-422), 9 (533-551), 11 (639-646)

SRC (NM_005417) 14 (530-537)

STK11 (NM_000455) 1 (36-77), 4-5 (193-211), 6 (261-288), 8 (332-370)

TP53 (NM_000546) 2 (1-12), 4 (69-112), 5-7 (126-253), 8 (267-306), 10 (332-342)

VHL (NM_000551) 1 (88-114), 2 (129-155), 3 (157-200)

XPO1 (NM_003400) 14-15 (501-575)

**Table 1C. 28 gene panel**

**Gene Exons (codons) tested**

ABL1 (NM_005157) 1-10 (1-523), 11 (560-638), 11 (661-1131)

ASXL1 (NM_015338) 2-12 (20-1542)

BRAF (NM_004333) 2-11 (47-465), 12-17 (478-709), 18 (729-767)

DNMT3A (NM_022552) 2-3 (1-59), 4-6 (89-196), 7-8 (214-322), 9-20 (339-803), 22-23 (827-913)

EGFR (NM_005228) 1-28 (1-1133), 28 (1171-1211)

EZH2 (NM_004456) 2-20 (1-752)

FLT3 (NM_004119) 2-24 (15-994)

GATA1 (NM_002049) 2-3 (1-182), 4-6 (200-336)

GATA2 (NM_032638) 2 (1-20), 3 (77-140), 3 (181-220), 3 (242-291), 4-5 (318-381), 6 (399-481)

HRAS (NM_005343) 2-4 (18-129), 5 (151-190)

IDH1 (NM_005896) 3 (1-13), 4-10 (41-415)

IDH2 (NM_002168) 1-7 (1-323), 9-11 (361-453)

IKZF2 (NM_016260) 2-8 (1-527)

JAK2 (NM_004972) 3-7 (11-220), 7-17 (241-723), 17-25 (747-1133)

KIT (NM_000222) 1-16 (1-765), 17-21 (788-977)

KMT2A (NM_005933) 2-3 (145-456), 3-6 (476-1212), 7-12 (1218-1507), 13-22 (1526-1979), 23 (1985-2024), 24-27 (2032-2690), 27 (2710-2747), 27-36 (2798-3970)

KRAS (NM_004985) 2-5 (1-189)

MDM2 (NM_002392) 1-5 (1-120), 7-11 (143-498)

MPL (NM_005373) 1-3 (1-122), 4-9 (131-490), 11-12 (522-636)

MYD88 (NM_002468) 1-5 (10-310)

NOTCH1 (NM_017617) 1-3 (1-135), 4 (197-237), 5-6 (248-367), 8-11 (419-587), 11-12 (632-672), 13-14 (716-785), 16-17 (823-885), 18 (914-981), 19 (1034-1057), 21-22 (1109-1196), 23 (1215-1294), 24 (1304-1338), 25-26 (1373-1673), 27 (1701-1723), 28 (1737-1779), 29-31 (1795-1935), 31 (1965-1978), 32-34 (2012-2556)

NPM1 (NM_002520) 1-11 (1-295)

NRAS (NM_002524) 2 (1-37), 3-5 (77-190)

PTPN11 (NM_002834) 1-8 (1-299), 9-15 (312-594)

RUNX1 (NM_001754) 3-6 (20-205), 8-9 (269-435), 9 (460-481)

TET2 (NM_001127208) 3 (1-616), 3-11 (636-2003)

TP53 (NM_000546) 2 (1-25), 4-6 (41-224), 7-10 (234-367)

WT1 (NM_024426) 1 (25-104), 1-2 (126-257), 4-10 (291-518)

Note: Coverage by gene and codon(s) tested for adequate amplicons

| **Supplementary Table 2. List of chemotherapy regimens on and off protocol** | | | |
| --- | --- | --- | --- |
| **MDA Protocol and NCT numbers** | **Regimen**  **Type** | **Drugs** | **Number of patients (N=87)** |
| 2010-0374 (NCT01202877) | Doublet | Azacitidine + Midostaurin | 7 |
| 2010-0511 (NCT01254890) | Doublet | Azacitidine + Sorafenib | 10 |
| 2012-1047 (NCT01892371) | Doublet | Quizartinib + LDAC or Azacitidine | 16 |
| 2014-0076 (NCT02196857) | Doublet | Azacitidine + Sorafenib | 11 |
| 2017-0912 (NCT03404193) | Triplet | Decitabine + Venetoclax + Sorafenib or Gilteritinib | 11 |
| 2018-0394 (NCT03661307) | Triplet | Decitabine + Quizartinib + Venetoclax | 4 |
| 2019-0366 (NCT04140487) | Triplet | Azacitidine + Gilteritinib + Venetoclax | 6 |
| Outside of a protocol | Doublet | Azacitidine or Decitabine + Sorafenib | 13 |
| Outside of a protocol | Doublet | Cladribine +LDAC + Sorafenib | 2 |
| Outside of a protocol | Doublet | LDAC + Sorafenib | 1 |
| Outside of a protocol | Triplet | Decitabine + venetoclax + sorafenib, or midostaurin, or gilteritinib | 6 |

Doublet, low-intensity chemotherapy + FLT3 inhibitor; Triplet, low-intensity chemotherapy + FLT3 inhibitor + venetoclax; NCT, national clinical trial identification number; LDAC, low dose cytarabine

| **Supplementary Table 3. Low Intensity Chemotherapy Backbones and FLT3 Inhibitors** | | | |
| --- | --- | --- | --- |
|  | **Doublet**  **N= 60**  **N (%) [range]** | **Triplet**  **N= 27**  **N (%) [range]** | ***P value*** |
| **LIC backbone** |  |  |  |
| Azacitidine | 46 (77) | 6 (22) | **<0.01** |
| Decitabine | 4 (6) | 21 (78) |  |
| LDAC | 8 (14) | 0 (0) |  |
| Cladribine/LDAC | 2 (3) | 0 (0) |  |
| **FLT3 inhibitor** |  |  |  |
| Sorafenib | 36 (60) | 10 (37) | **<0.01** |
| Midostaurin | 8 (13) | 1 (4) |  |
| Quizartinib | 16 (27) | 4 (15) |  |
| Gilteritinib | 0 (0) | 12 (44) |  |
| Venetoclax, yes | 0(0) | 27 (100) | n/a |
| **ASCT in CR1** | 6 (10) | 8 (30) | **0.02** |

Doublet, low-intensity chemotherapy + FLT3 inhibitor; Triplet, low-intensity chemotherapy + FLT3 inhibitor + venetoclax; LIC, low intensity chemotherapy; LDAC, low dose cytarabine; ASCT, allogeneic stem cell transplant; CR1, first remission

| **Supplementary Table 4. End of cycle 1 responses and count recovery** | | | | |
| --- | --- | --- | --- | --- |
| **End of Cycle 1** | **Doublet** | **# Pts Evaluable** | **Triplet** | **# Pts Evaluable** |
| **Response** |  |  |  |  |
| CR | 7 (11) | 60 | 13 (48) | 27 |
| CRi | 19 (32) | 60 | 10 (37) | 27 |
| CR/CRi | 26 (43) | 60 | 23 (85) | 27 |
| MFC negative | 12 (46) | 26 | 13 (62) | 21 |
| FLT3 PCR negative | 5 (20) | 25 | 15 (68) | 22 |
| **Count recovery** |  |  |  |  |
| ANC >500, median day | 21 [7-57] | 16 | 40 [24-54] | 20 |
| PLT >50, median day | 25 [6-72] | 14 | 29 [15-48] | 21 |
| ANC, absolute neutrophil count, PLT, platelet; MFC, multicolor flow cytometry; CR complete response; CRi CR with incomplete count recovery | | | | |
|  |  |  |  |  |

**Supplementary Table 5. Cox regression model – overall survival**

| **Variables** | **Sig.** | **Exp(B)** | **95.0% CI for Exp(B)** | |
| --- | --- | --- | --- | --- |
|  |  |  | **Lower** | **Upper** |
| **Doublet vs. Triplet** | 0.024 | 3.277 | 1.167 | 9.202 |
| **FLT3-ITD Allelic Ratio (High vs. Low)** | 0.287 | 0.721 | 0.395 | 1.316 |

**Supplementary Figure 1. Detailed Dose Schedules of Doublet (Blue) and Triplet (Green) Regimens**


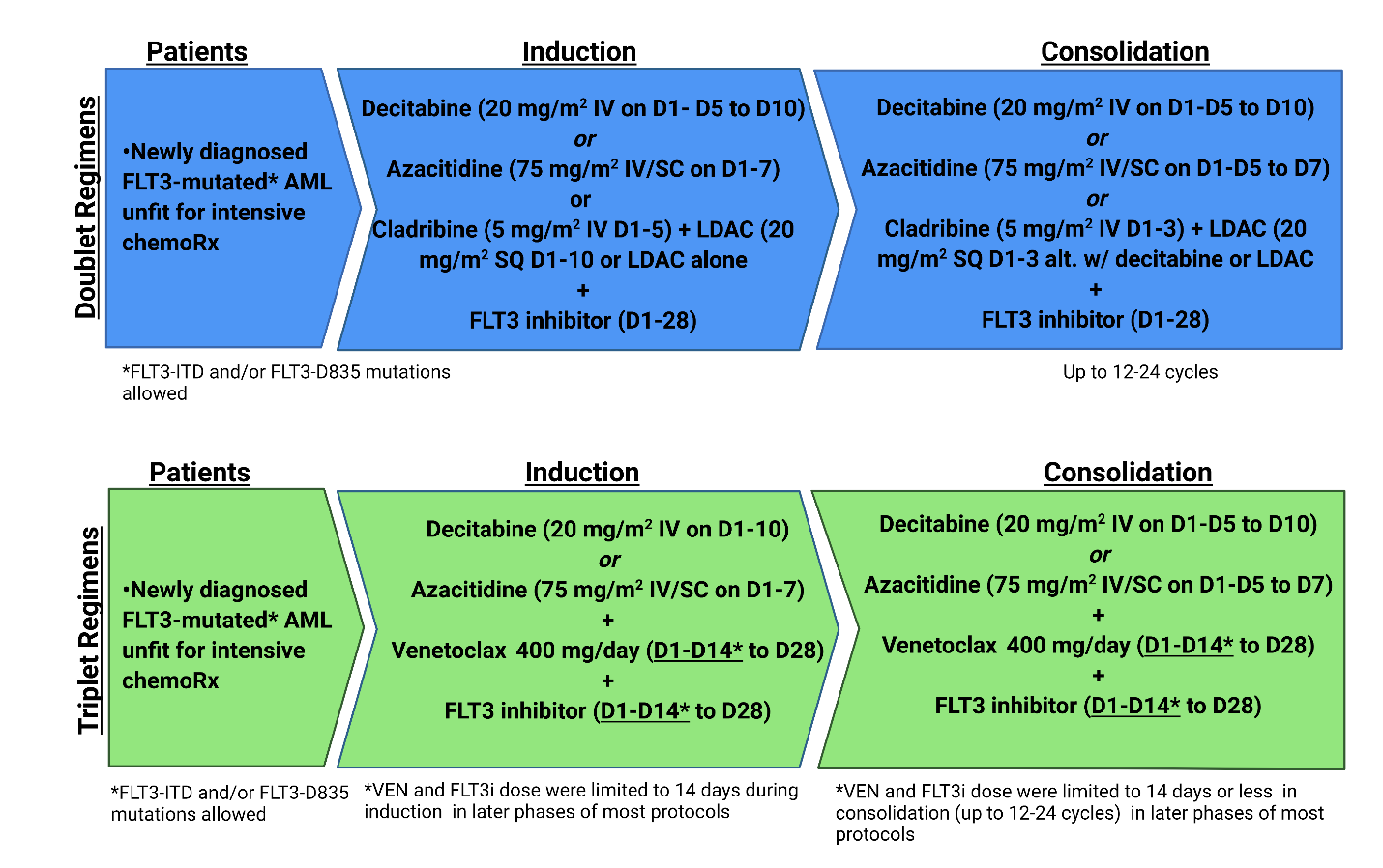


*This figure describes the chemotherapy regimens used in this study. The doublet regimens are summarized in the blue figure. During induction - Decitabine given 20 mg/m² from day 1-5 or to day 10 or Azacitidine 75 mg IV or subcutaneous on day 1-7 or Cladribine given as 5 mg/m² IV from day 1-5+ low-dose cytarabine or low-dose cytarabine alone. FLT3 inhibitors are administered on a daily basis beginning on day 1. FLT3i dose ranges are as follows: sorafenib 200-400 mg twice a day, midostaurin 75 mg twice a day, quizartinib 30-40 mg daily, and gilteritinib 80-120 mg daily. Doses of chemotherapy during consolidation are, as shown. The triplet regimens are summarized in the green figure. Decitabine or azacitidine given in a dose schedule similar to that shown in the doublet schedule. In some of the protocols, venetoclax was initially administered at a dose of 400 mg daily for up to 28 days. However, due to prolonged myelosuppression, the VEN and FLT3 inhibitors were subsequently limited to 14 days in cycle 1 in patients who achieve <5% blasts or marrow hypo/aplasia by C1D14. Similarly, in earlier stages of these protocols, FLT3 inhibitors were typically administered for 28 days during induction but in more recent amendments of the protocol have been reduced to 14 days in cycle 1 in patients who achieve <5% blasts or marrow hypo/aplasia by C1D14 . Doses of chemotherapy during consolidation are, as shown.* Overall, p*atients received median 3 cycles of therapy (range 1-35 cycles).*

**Supplementary Figure 2. Relapse Free Survival – Triplet versus 1^st^ generation FLT3i doublets versus 2^nd^ generation FLT3i doublets**

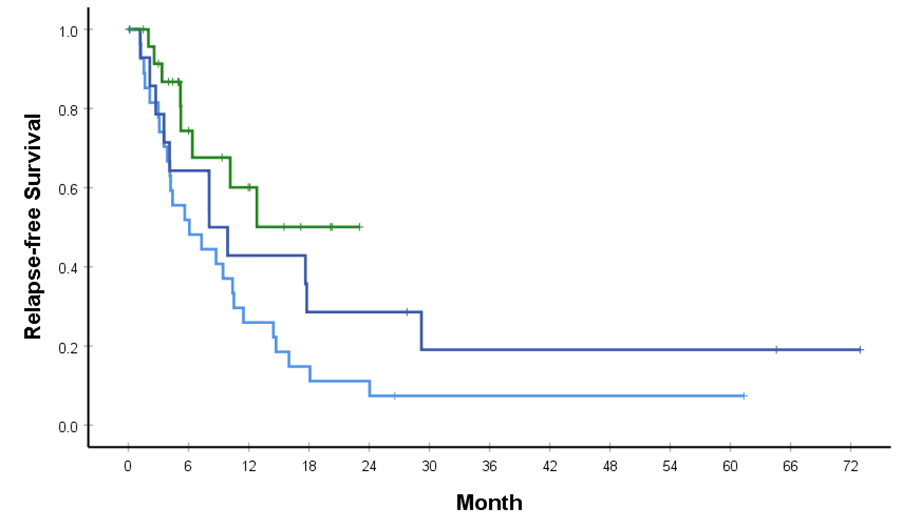


**Supplementary Figure 3A. Overall Survival of Patients Treated with a Triplet Regimen Incorporating a First Generation versus A Second Generation FLT3 Inhibitor**

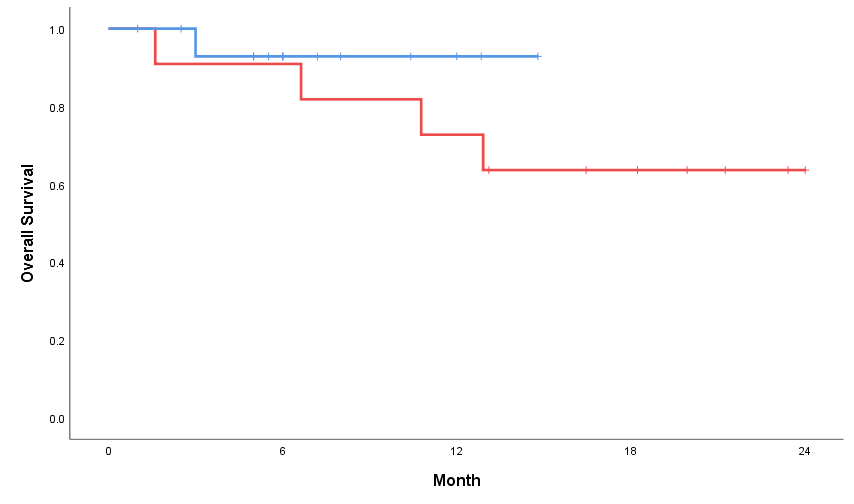


**P=0.37**

**Supplementary Figure 3B. Overall Survival of Patients Treated with a Triplet or a Doublet Regimen Incorporating a First Generation versus a Second Generation FLT3 Inhibitor**

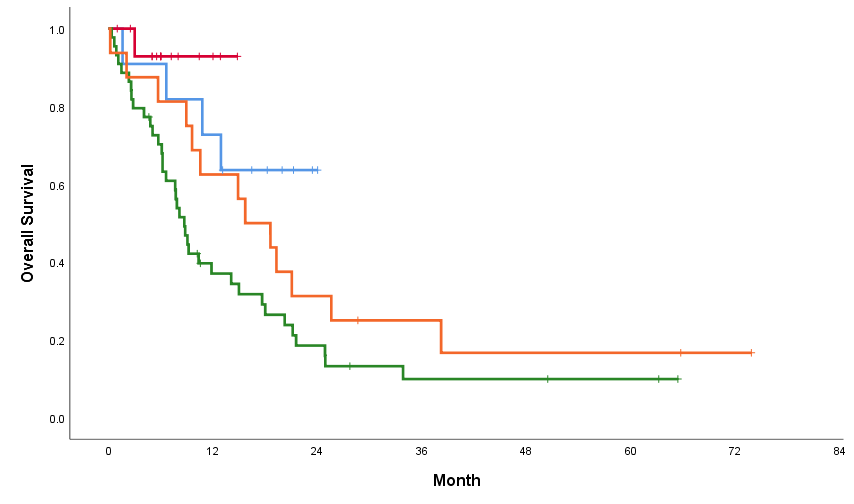


**P=0.01**

LIC, low intensity chemotherapy; FLT3i, FLT3 inhibitor; NR, not reached; OS, overall survival

**Supplementary Figure 4. The impact of FLT3-ITD AR (high [≥0.5] vs. low [<0.5]) on OS by therapy**
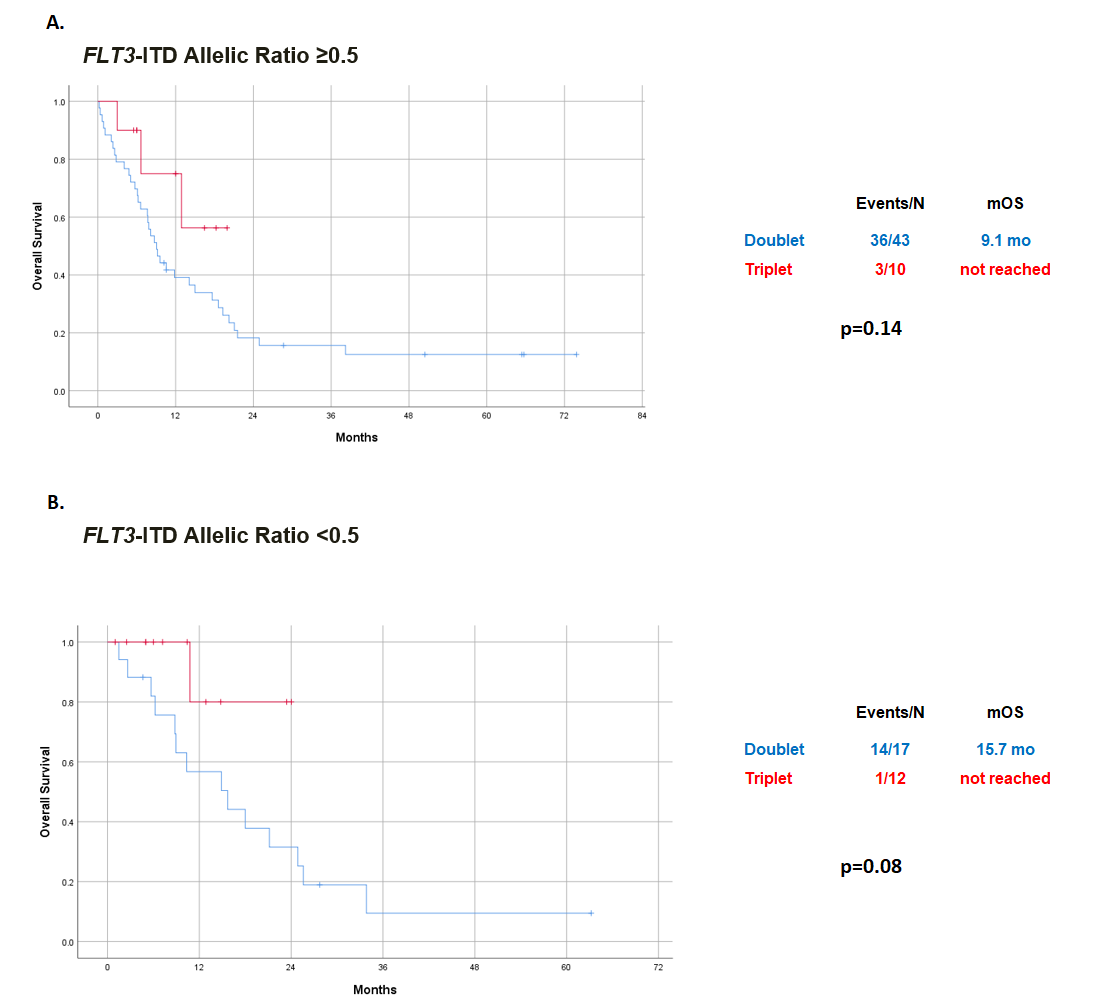


**Supplementary Figure 5A. Overall Survival by MFC based Measurable Residual Disease (MRD) Status in all CRc patients**

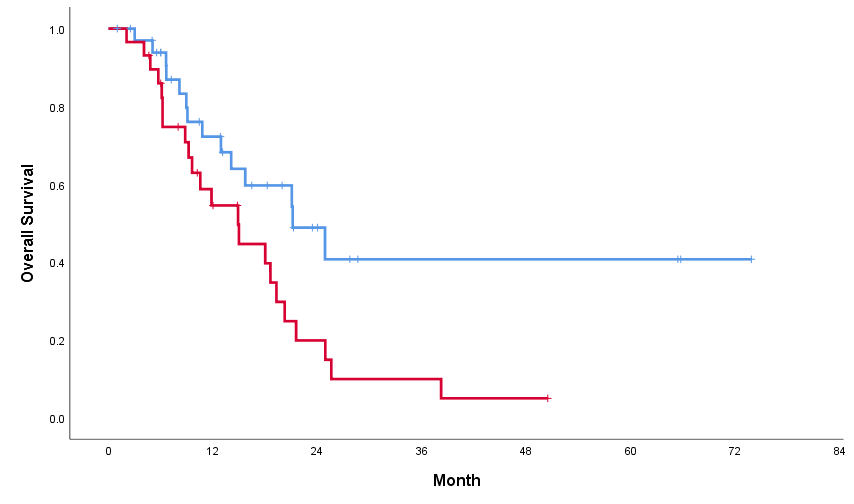


**P=0.02**

**Supplementary Figure 5B. Overall Survival by PCR based Measurable Residual Disease (MRD) Status in all CRc patients**

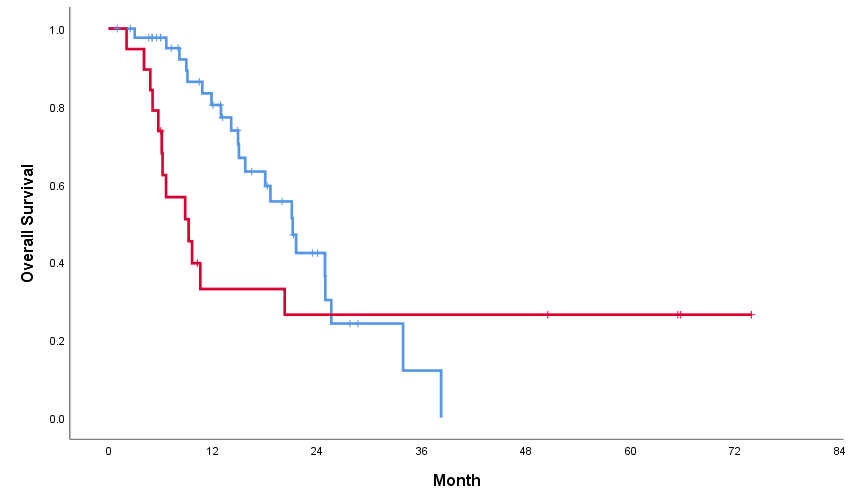


**P=0.19**

PCR, RT-polymerase chain reaction assay for FLT3; MFC, multicolor flow cytometry; OS, overall survival

**Supplementary Figure 6A. Landmark Analysis for ASCT**


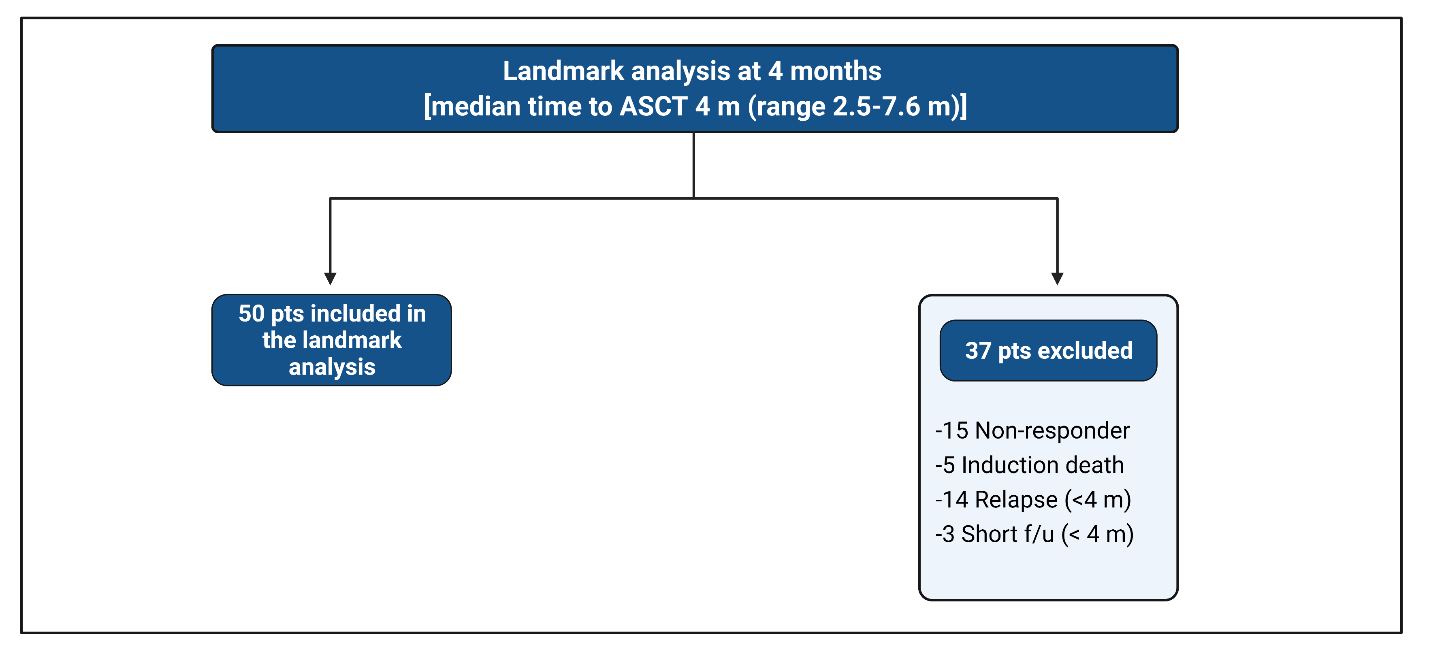


Pts, Patients; ASCT, allogeneic stem cell transplant; f/u, follow-up

**Figure 6B. Overall Survival Rates by Allogeneic Stem Cell Transplantation in First Remission – A landmark Analysis**


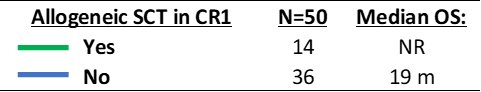


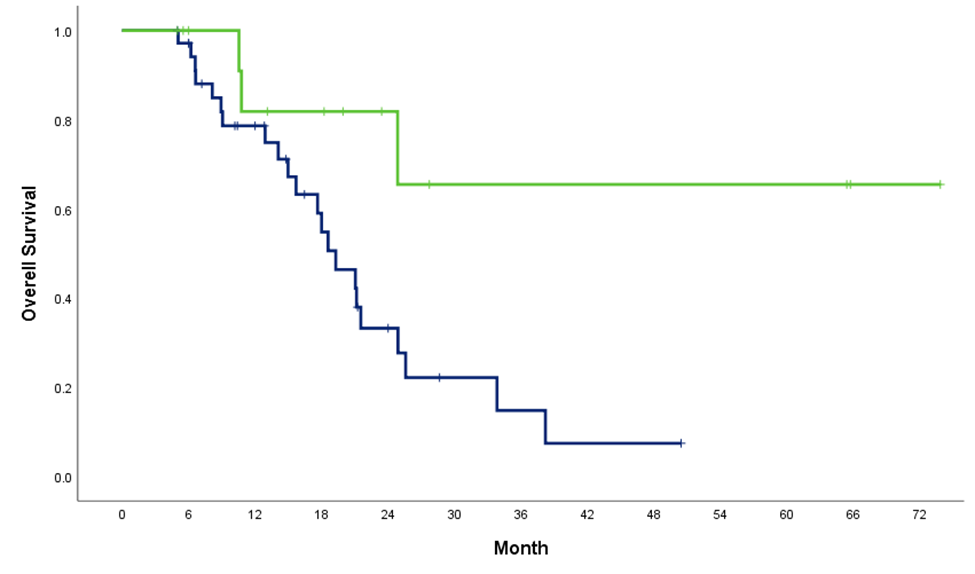


**P=0.01**

**Supplementary 6C. Overall Survival Rates by Allogeneic Stem Cell Transplantation in CR1 – A landmark Analysis only for patients treated in the Doublet Arm (N=29)**


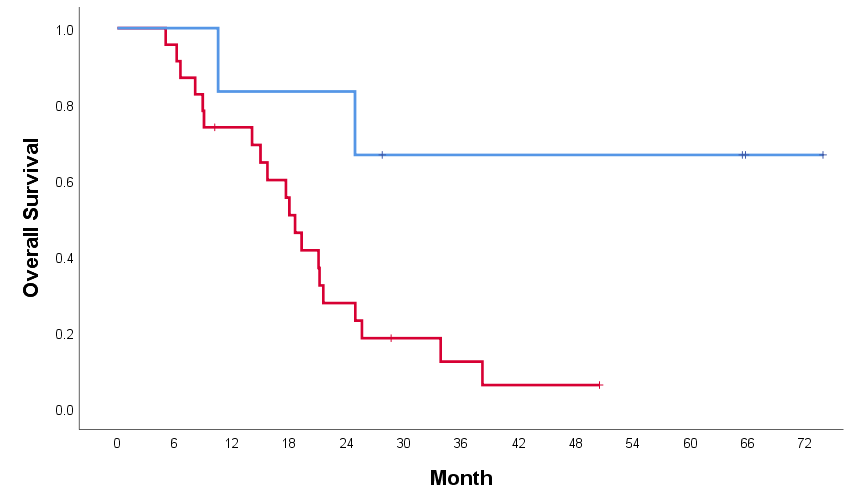


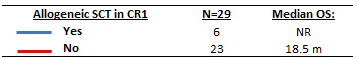


**Supplementary 6D. Overall Survival Rates by Allogeneic Stem Cell Transplantation in First remission – A landmark Analysis only for patients treated in Triplet Arm (N=21)**


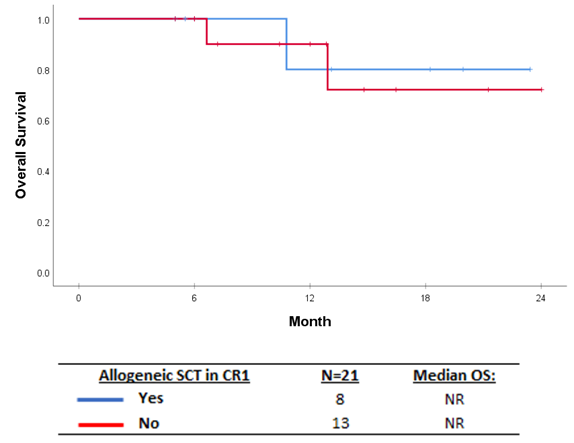

Supplement: Supplementary file 1 — Supplemental Tables and Figures [file 41408_2022_670_MOESM1_ESM.docx]
